# Disguised Emotion in Alexithymia: Subjective Difficulties in Emotion Processing and Increased Empathic Distress

**DOI:** 10.3389/fpsyt.2020.00698

**Published:** 2020-07-17

**Authors:** Gieun Nam, Hyerin Lee, Jang-Han Lee, Ji-Won Hur

**Affiliations:** ^1^ Clinical Neuro-Psychology Lab, Department of Psychology, Chung-Ang University, Seoul, South Korea; ^2^ Department of Psychology, Chung-Ang University, Seoul, South Korea; ^3^ Department of Psychology, Korea University, Seoul, South Korea

**Keywords:** alexithymia, emotion processing, empathy, personal distress, gender

## Abstract

Despite decades of speculation, many causal aspects that contribute to the heterogeneity of alexithymia still must be clarified. This study examined the extent of the alexithymia phenotype and its contribution to social function in the general population. In total, 200 participants (females = 111) completed the Toronto Alexithymia Scale-20 (TAS-20), multiple self-reporting questionnaires measuring emotion intelligence, empathy, hostility and impulsivity, and the Reading the Mind in the Eyes Test (RMET). In the multivariate analysis, highly alexithymic individuals appeared to report subjective deficits in emotion recognition and regulation as well as increased impulsivity; however, their empathy skills were intact, and even the proneness to experiencing empathic distress with others’ suffering was increased among alexithymic individuals. We also compared the clinical and behavioral manifestations of highly alexithymic male and female subjects to those of each gender control group. As a result, in contrast to their subjective self-reports of emotion processing impairment, the RMET performance appeared to be preserved in alexithymic females; however, highly alexithymic males showed actual deficits in the emotion identification task. Future research needs to further refine the constructs of alexithymia to incorporate the phenotypic changes in affected individuals in relation to measuring instruments, the extent of empathic distress, and gender.

## Introduction

Alexithymia (from the Greek stems, a = lack, lexis = word, and thymos = emotion, literally “lack of words for emotion”) was coined by Sifneos (1) to denote a stable, dimensional psychological construct that includes difficulties in identifying, describing, and distinguishing emotions and an externally oriented thought style (2, 3). Previous studies have shown that alexithymia exists on a continuum in the general population (4), and from 5 to 19% of the population has recently been found to have alexithymia (5, 6). Individuals with a high degree of alexithymia are reported to have more dysfunction in coping with daily stressors and to be more vulnerable to mental illnesses (7–10).

In the early years of research, these mental problems were known to be related to a reduced ability to recognize others’ emotions as well as to experience empathy. However, newly accumulated findings, to which various research methods have been applied, have begun to neutralize the hypothesis of social dysfunction in alexithymia (9). For instance, behavioral evidence has demonstrated that social cognition in individuals with alexithymia is quite intact (11–14). In accordance with this finding, emotion perception deficits in alexithymia, once considered relatively reliable features, have become controversial issues (15, 16).

In particular, the works of Freyberger (17) and McDougall (18) raised the possibility that alexithymia could be a consequence of an inefficient strategy against overwhelming affects rather than a primary deficit of emotional experience. These psychodynamic approaches provided a more complex perspective that alexithymia may be related to immature defense mechanisms such as repression and/or avoidance of intense emotional experiences (19–21). In addition, empirical evidence obtained using emotional or visceral stimuli have suggested relations between alexithymia and physiological hyperarousal (22–24). For instance, the most recent research using odor stimuli (25) and a case study (26) have provided evidence that highly alexithymic individuals who seem to have limited emotional experience might, in fact, have an improperly elevated physiological response (i.e., arousal) to experimental stimuli.

An equally incoherent picture has emerged from research exploring the relationships of alexithymia with problematic emotional expressions, including impulsivity, aggression, or hostility (27–32). For example, while gender has been reported as a significant variable influencing impulse control problems such as drinking, gambling, and suicide (33), little is known about whether gender contributes to alexithymia-related externalizing problems.

Indeed, gender is another issue of particular importance in alexithymia. Early research about this subclinical phenomenon was originally intended to illuminate the male-specific altered emotional profile: Levant (34) suggested the *normative male alexithymia hypothesis*. Levant believed that males under the pressure of gender socialization were discouraged from expressing feelings, namely, weakness or attachment feelings and that they consequently displayed alexithymia (34, 35). As research has continued, however, this undifferentiated emotional experience was found to not be exclusive to males (36–38). The early findings that alexithymia was more prevalent in males (34, 39, 40) have not been consistently replicated in subsequent studies (6, 41–44).

On the one hand, the mixed results in alexithymia research may partially stem from the research methods such as the nature of the clinical populations involved in the study or the self-questionnaire assessments (40). Indeed, many studies have been performed in such a way that self-reporting questionnaires are conducted for alexithymic subjects with various mental disorders such as depression, substance abuse, psychosomatic illnesses, or chronic pain (29, 30, 32). Therefore, there has been a need for general population-based research including behavioral experiments to generalize the clinical implications of alexithymia (45, 46).

In short, this study was designed to clarify the phenotypes of alexithymia. Considering the major methodological limitations in previous research, the aim of the present study was to examine whether highly alexithymic individuals in the general population have emotion processing deficits, including problems with (a) emotion recognition, (b) emotion regulation, and (c) empathy as well as to explore the salient gender-specific features of alexithymia. Multivariate analysis of covariance (MANCOVA) was also used to compare the different clinical features within each gender group. In particular, we assumed that considering gender in alexithymia research may shed light on understanding alexithymia-related emotions and externalizing behaviors (47). Therefore, we compared the behavioral and clinical manifestations of alexithymic subjects to gender control subjects in order to identify unique clinical phenotypes in highly alexithymic male or female subjects. In addition, regarding the limitations of previous research using self-report questionnaires (48), we also used a four-alternative forced-choice paradigm, the Reading the Mind in the Eyes Test (RMET) (49, 50) to objectively assess the ability to decode others’ emotional experiences in individuals with alexithymia. We expect that the current research will help to comprehend the extent of the alexithymia phenotype and its implications that have previously been contradictory.

## Method

### Participants

The participants included 200 individuals aged between 19 and 32 years who agreed to participate in the study. Korean was the first language of participants, and none of them had a problem communicating or responding to the questionnaires. All participants had normal or corrected-to-normal visual acuity. We placed advertisements in an Internet community and on a Social Network System to recruit research participants with diverse ages and educational levels. This study was approved by the University Ethics Committee (1041078-201707-BRSB-148-01). All participants provided informed consent.

### Materials

Five questionnaires and one experimental task were used in this study to measure the participants’ alexithymic tendency, emotional intelligence, and multiple dimensions of empathy, impulsivity, hostility, and behavioral empathy ability. The entire procedure took 50 min on average.

#### Korean Version of the 20-Item Toronto Alexithymia Scale (TAS-20K)

The alexithymic traits were measured by the Toronto Alexithymia Scale (TAS-20K) (51–53). This scale consists of 20 items rated on a 5-point Likert scale according to the symptom severity. TAS-20K consists of three factors: (1) difficulty identifying feelings (DIF). Seven items are used to identify emotions and the emotional distinction between emotional and physical senses. (2) Difficulty describing feelings (DDF). This factor indicates the ability to express subjective emotions to others and consists of five items. (3) Externally oriented thinking (EOT). This factor consists of eight items that assess the ability to express externally oriented thinking. The internal consistency for the original scale was reported to be 0.81 (53). Cronbach’s alpha for this study was 0.82.

#### Emotional Quotient

The individual ability to process emotional experience was assessed by the emotional quotient (EQ) (54, 55). This tool consists of five subscales: perception and expression of emotion, empathy, integrate emotion to facilitate thought, use of emotions, and regulation of emotions. A total of 50 items (10 items per subscale) are rated on a 5-point Likert scale, and a higher score indicates that an individual has higher emotional intelligence. Cronbach’s alpha of the adult emotional intelligence test was 0.96 (55). In this study, Cronbach’s alpha of EQ was 0.84.

#### Korean Version of the Interpersonal Reactivity Index

Multiple dimensions of empathy were measured by the Interpersonal Reactivity Index (IRI) (56, 57) which consist of four separated dimensions of empathy. This measure is a self-report questionnaire using a 5-point Likert scale consisting of 28 items and two factors. The first factor, cognitive empathy, consists of perspective taking (7 items: the tendency to immediately adopt the viewpoint of others) and fantasy (7 items: the tendency to imaginatively transpose oneself into the feelings and actions of fictitious characters). The second factor, emotional empathy, consists of empathic concern (7 items: the tendency to experience feelings of sympathy and concern for others’ misfortune and for their welfare) and personal distress (7 items: self-directed feelings of anxiety, discomfort, and unpleasantness in response to tense interpersonal situations). The internal consistency of the scale was 0.80, and the test-retest reliability was 0.76 (57). In this study, Cronbach’s alpha of IRI was 0.77.

#### Buss-Durkee Hostility Inventory

Hostility was assessed by the Buss-Durkee Hostility Inventory (BDHI). The subscales measuring active aggressiveness were extracted from the original Hostility Inventory (58, 59) to determine externalized expression in alexithymic individuals. A total of 21 items were rated with a 4-point Likert scale. The internal consistency of this scale was 0.78, and the test-retest reliability was 0.79 in undergraduate students (59). In the present study, the internal consistency was 0.85.

#### Barratt Impulsiveness Scale-11-Revised

Impulsivity was assessed by the Barratt Impulsiveness Scale-11-Revised (BIS). This scale was first developed by Barratt and has since been re-established as the BIS-11 (60–62). The BIS-11 consists of 30 self-report questionnaires, including 11 revised scoring items, and each question is rated with a 4-point Likert scale. The score is calculated by summing the scores of each item considering the negatively scored items. A higher total score indicates greater impulsivity. The internal consistency coefficient (Cronbach’s alpha) of the Korean version was 0.78 (61). Cronbach alpha of BIS in the current sample was 0.81.

#### Korean Version of the Reading the Mind in the Eyes Test (RMET)

Based on a photograph of a person’s eyes, the participant must choose the most plausible answer to a question regarding the person’s feelings and intentions (49, 50). This task is used to assess the individual’s ability to perceive emotions as a basic function of social cognitive function. The RMET consists of 36 photographs, each of which show a photograph of a face with a rectangle cut out to display only the eye. The photographs are 5 x 2 inches in size and are displayed in monochrome. Four target words are presented (one correct word and three foils) with a picture of the eye. Each of the four words is presented at the four corners of the rectangle, with a number immediately below the word, allowing participants to select the word by pressing a number. There was no time limit for choosing words. The presentation and experimental stimulation were carried out using the PsychoPy program (63), which is free software program that can create and execute psychological experiments for the production and implementation of RMET tasks.

### Procedure

When participants arrived at the laboratory, they first received an explanation of the overall study and agreed to study participation. Participants who signed the agreement to participate were given a brief explanation of the experimental computer operation by listening to the explanation of the research process. Then, self-report questionnaires, including demographic data questionnaires, were conducted through computer-generated screens. The RMET task was conducted after an explanation of how to perform the task was provided by the researcher.

When the RMET task started, the first page displayed a brief description of the assignment, along with the explanations on the page. After participants were instructed to choose only one word that best matched the stimulus presented, they proceeded to the next page and performed a practice item and then the full-scale experiment. The experiment was completed by conducting a debriefing of the experiment. The total duration of the RMET experiment was approximately 20 min, and no break was given during the test.

### Statistical Analysis

We compared the demographic characteristics of alexithymic participants and non-alexithymic participants. In this study, we used a binary cutoff and divided participants as alexithymic (TAS-20K total score ≥ 52) and non-alexithymic (TAS-20K total score ≤ 51) according to previous studies that suggested that a TAS-20K total score ≥ 52 indicates the presence of moderate alexithymia (64–66). Frequency analyses and descriptive statistics were used to examine demographic characteristics such as the age and education level of participants. The primary comparisons of RMET scores and clinical measures were conducted using MANOVA.

The data were also divided by gender in order to assess potential gender differences. Thus, a MANCOVA test with age as a covariate as well as correlation analyses were used to assess gender-specific features of alexithymia. Then, multivariate tests of the simple effects of gender group differences were conducted to identify the behavioral and clinical manifestations of highly alexithymic individuals within each gender group. Regarding previous research (65, 67), the highest 25^th^ percentile (alexithymic males ≥ 52; alexithymic females ≥ 58 total TAS-20K score) was defined as the alexithymia group, and the lowest 25^th^ percentile was defined as the non-alexithymic control group (non-alexithymic males ≤ 38; non-alexithymic females ≤ 42 total TAS-20K score). The statistical analysis was conducted using SPSS 25.0 for Windows (SPSS Inc.; Chicago, IL, USA).

## Results

### Alexithymia vs. Control Groups for All the 200 Participants

#### Demographic Characteristics

Among our participants, 89 (44.5%) were male, and 111 (55.5%) were female, resulting in a higher percentage of female participants. The mean age of the all participants was 23.07 years (*SD* = 2.68), and male participants were significantly older than females (*t*(198) = 3.78, *P* <.001). There were no significant group differences in education level (*χ²*(3) = 5.73, *P* = .13) (**Table 3**).

Of the 200 participants, 71 (14.2%) were classified as having alexithymia according to the previously recommended cutoff score. There were no significant differences in age and education level (*t*(198) = 1.49, *P* = .14 and *χ²*(3) = 0.90, *P* = .83, respectively) between the alexithymia (≥52 on the TAS-20K determined at screening) and non-alexithymia groups (**Table 1**).

**Table 1:** Group comparison of demographic characteristics by alexithymic tendency.

	Alexithymic(n = 71)	Non-alexithymic(n = 129)	Statistics	*P*
Age, mean (SD)	22.69 (2.74)	23.28 (2.62)	*t*(198) = 1.49	.137
Education			*χ*²(3) = 0.90	.825
High school graduate or lower, n	12	17		
Undergraduate student, n	43	77		
University graduate, n	15	32		
Graduate school or higher, n	1	3		
Toronto Alexithymia Scale-20K	(Wilks’ lambda = 0.32, ***F*_(3, 196)_ = 137.71, *P* <.001**, *η^2^_p_* = .68)			
Difficulty identifying feelings	22.20 (4.77)	13.22 (3.78)	**213.48**	**<.001**
Difficulty describing feelings	17.34 (3.53)	10.64 (2.70)	**224.83**	**<.001**
Externally oriented thinking	19.76 (2.95)	17.10 (3.05)	**35.63**	**<.001**
Total score	59.30 (5.82)	40.96 (6.38)	**402.03**	**<.001**

bold: statistically significant.

#### Behavioral and Clinical Characteristics

MANOVA was used to determine whether there was a significant difference in dependent variables (RMET, EQ, IRI, BDHI, BIS scores) between alexithymic and non-alexithymic participants (Wilks’ lambda = 0.78, *F*_(12, 187)_ = 4.51, *P* <.001, *η^2^_p_* = .22) (**Table 2**). In this analysis, we demonstrated that there was a significant decrease in most of the EQ subscales (EQ perception and expression of emotion: *F*_(1,198)_ = 31.64, *P* <.001, *η^2^_p_* = .14; EQ integration of emotion to facilitate thought: *F*_(1,198)_ = 5.61, *P* = .02, *η^2^_p_* = .03; EQ use of emotions: *F*_(1,198)_ = 5.53, *P* = .02, *η^2^_p_* = .03; EQ regulation of emotions: *F*_(1,198)_ = 12.74, *P* <.001, *η^2^_p_* = .06) and IRI subscales (IRI perspective taking, *F*_(1,198)_ = 4.16, *P* = .04, *η^2^_p_* = .02; and IRI higher personal distress, *F*_(1,198)_ = 21.84, *P* <.001, *η^2^_p_* = .10) for the alexithymia groups. Alexithymic individuals also showed increased impulsivity (BIS, *F*_(1,198)_ = 10.42, *P* = .00, *η^2^_p_* = .05). However, there were no significant group differences in RMET performance (*F*_(1,198)_ = 2.26, *P* = .14, *η^2^_p_* = .01) or hostility (BDHI, *F*_(1,198)_ = 0.18, *P* = .68, *η^2^_p_* = .00).

**Table 2:** Comparison of measurements in alexithymic and non-alexithymic participants.

	Alexithymic(*n* = 71)	Non-alexithymic(*n* = 129)	*F*	*P*	*η_p_^2^*
RMET	26.14 (2.77)	26.81 (3.12)	2.26	.135	.01
EQ					
* Perception and expression of emotion*	34.90 (5.06)	38.56 (3.99)	**31.64**	**<.001**	**.14**
* Empathy*	37.69 (4.80)	37.58 (4.46)	0.03	.873	.00
* Integrate emotion to facilitate thought*	35.15 (5.22)	36.87 (4.71)	**5.61**	**.019**	**.03**
* Use of emotions*	33.86 (4.32)	35.26 (3.84)	**5.53**	**.020**	**.03**
* Regulation of emotions*	31.46 (5.47)	34.26 (5.21)	**12.74**	**<.001**	**.06**
IRI					
* Perspective taking*	17.93 (3.75)	19.13 (4.11)	**4.16**	**.043**	**.02**
* Fantasy*	17.46 (5.39)	18.02 (5.23)	0.51	.475	.00
* Empathy concern*	17.77 (4.11)	17.82 (4.61)	0.01	.943	.00
* Personal distress*	16.21 (4.80)	12.56 (5.54)	**21.84**	**<.001**	**.10**
BDHI	44.90 (9.37)	45.48 (9.32)	0.18	.675	.00
BIS	67.27 (10.16)	62.73 (9.14)	**10.42**	**.001**	**.05**

mean (SD); bold: statistically significant.

RMET, Reading the Mind in the Eyes Test; EQ, emotional quotient; IRI, Interpersonal Reactivity Index; BDHI, Buss-Durkee Hostility Index; BIS, Barratt Impulsiveness Scale-11-Revised.

### Gender-Related Behavioral and Clinical Features in Alexithymia

#### Differences in Alexithymia by Gender

There were no significant gender differences in education level, but age differences were found (males 23.8 ± 2.5 years vs. females 22.5 ± 2.7, *t*_(198)_ = 3.78, *P* <.001). MANCOVA was used to assess the TAS-20K of the two gender groups adjusted by subject age (as a covariate) (Wilk’s lambda = 0.95, *F*_(3,195)_ = 3.78, *P* = .01, *η^2^_p_* = .05).

As a result, the TAS-20K total score was significantly higher for female participants than for males (*F*_(1,197)_ = 5.09, *P* = .03, *η^2^_p_* = .03). We found that there were significantly elevated scores in the TAS-20K DIF factor (*F*_(1,197)_ = 9.34, *P* = .00, *η^2^_p_* = .05) in female subjects, and the TAS-20K DDF factor (*F*_(1,197)_ = 3.54, *P* = .06, *η^2^_p_* = .02) among female subjects compared to males was marginally increased. However, no significant gender difference was observed for the TAS-20K EOT factor (*F*_(1,197)_ = 0.39, *P* = .54, *η^2^_p_* = .00) (**Table 3**).

**Table 3:** Group comparison of demographic characteristics by gender.

	Male(*n* = 89)	Female(*n* = 111)	Statistics	
			*t*/*χ*²	*P*
Age, mean (*SD*)	23.84 (2.48)	22.45 (2.67)	***t*(198) = 3.78**	**<.001**
Education			*χ*²(3) = 5.73	.126
High school graduate or lower, *n*	7	22		
Undergraduate student, *n*	57	63		
University graduate, *n*	23	24		
Graduate school or higher, *n*	2	2		
Toronto Alexithymia Scale-20K	(Wilks’ lambda = 0.95, ***F*_(3,195)_ = 3.78, *P* = .011**, *η^2^_p_* = .05, covariate: age)			
* Difficulty identifying feelings*	14.72 (5.02)	17.76 (6.36)	**9.34**	**.003**
* Difficulty describing feelings*	12.27 (3.93)	13.62 (4.68)	3.54	.061
* Externally oriented thinking*	18.19 (3.32)	17.93 (3.24)	0.39	.535
Total score	45.18 (9.81)	49.31 (11.15)	**5.09**	**.025**

bold: statistically significant.

#### Correlation Analysis in Each Gender Group

The results of correlation analysis with the TAS-20K score and other measurements in male and female subjects are shown in **Tables 4** and **5**. Male participants showed a negative correlation in the TAS-20K total score, TAS-20K DIF and TAS-20K EOT with behavioral tasks and RMET (*r* = −.25, *P* <.05; *r* = −.29, *P* <.01; *r* = −.21, *P* <.05). However, in female subjects, there were no significant correlations in TAS-20K scores and RMET performance.

**Table 4:** Correlation analysis results with TAS-20K and other measurements in male participants.

	TAS Total	TAS DIF	TAS DDF	TAS EOT
RMET	−.25^*^	−.29^**^	−.09	−.21^*^
EQ								
Perception and expression of emotion	−.53^**^	−.36^**^	−.51^**^	−.43^**^
Empathy	−.04	.06	−.04	−.16
Integrate emotion to facilitate thought	−.38^**^	−.27^*^	−.36^**^	−.28^**^
Use of emotions	−.27^*^	−.12	−.26^*^	−.29^**^
Regulation of emotions	−.43^**^	−.35^**^	−.30^**^	−.38^**^
IRI								
Perspective taking	−.25^*^	−.13	−.17	−.32^**^
Fantasy	−.03	.09	−.11	−.11
Empathy concern	−.07	.00	−.05	−.14
Personal distress	.52^**^	.46^**^	.39^**^	.38^**^
BDHI	.22^*^	.24^*^	.04	.24^*^
BIS	.42^**^	.36^**^	.16	.49^**^

^*^P <.05; ^**^P <.01; bold: statistically significant.

TAS, Toronto Alexithymia Scale; DIF, difficulty identifying feeling; DDF, difficulty describing feeling; EOT, externally oriented thinking; RMET, Reading the Mind in the Eyes Test; EQ, Emotional Quotient; IRI, Interpersonal Reactivity Index; BDHI, Buss-Durkee Hostility Index; BIS, Barratt Impulsiveness Scale-11-Revised.

**Table 5:** Correlation analysis results with TAS-20K and other measurements in female participants.

	TAS Total	TAS DIF	TAS DDF	TAS EOT
RMET	−.13	−.14	−.03	−.14
EQ				
Perception and expression of emotion	−.54^**^	−.34^**^	−.58^**^	−.34^**^
Empathy	−.13	.02	−.20^*^	−.20^*^
Integrate emotion to facilitate thought	−.22^*^	−.21^*^	−.15	−.14
Use of emotions	−.33^**^	−.18	−.29^**^	−.36^**^
Regulation of emotions	−.32^**^	−.32^**^	−.21^*^	−.17
IRI				
Perspective taking	−.15	−.18	−.03	−.13
Fantasy	−.21^*^	−.10	−.22^*^	−.20^*^
Empathy concern	−.10	.01	−.12	−.19
Personal distress	.46^**^	.54^**^	.30^**^	.09
BDHI	.03	.04	−.04	.07
BIS	.25^**^	.28^**^	.08	.19^*^

^*^P <.05; ^**^P <.01; bold: statistically significant.

TAS, Toronto Alexithymia Scale; DIF, difficulty identifying feeling; DDF, difficulty describing feeling; EOT, externally oriented thinking; RMET, Reading the Mind in the Eyes Test; EQ, Emotional Quotient; IRI, Interpersonal Reactivity Index; BDHI, Buss-Durkee Hostility Index; BIS, Barratt Impulsiveness Scale-11-Revised.

#### Alexithymic Male Group vs. Non-Alexithymic Male Group

Since we found significant gender differences in the patterns of alexithymia, potential differences between the alexithymic and non-alexithymic group were tested within each gender group by calculating MANOVAs using the RMET, EQ, IRI, BDHI, and BIS scores as the dependent measures. Male subjects who scored ≥ 52 on the TAS-20K, which was the highest 25^th^ percentile of TAS-20K scores for male participants, were assigned to the alexithymic group (*n* = 22). We assigned participants with the lowest 25^th^ percentile of TAS-20K scores (≤ 38 on the TAS-20K; *n* = 23) to the non-alexithymic group. We excluded the remaining participants with subthreshold symptoms of alexithymia from the multivariate analyses to rule out their possible effects on the dependent variables (**Table 6**).

**Table 6:** Comparison of measurements in alexithymic and non-alexithymic male participants.

	Alexithymic(*n* = 22)	Non-alexithymic(*n* = 23)	*F*	*P*	*η_p_^2^*
*[Wilks’ lambda = 0.35, **F_(12,32)_ = 4.85, P <.001**, η^2^_p_ = .65]*
RMET	26.05 (2.68)	27.87 (3.03)	**4.55**	**.039**	**.10**
EQ					
* Perception and expression of emotion*	35.73 (5.74)	41.35 (2.44)	**18.55**	**<.001**	**.30**
* Empathy*	38.32 (3.92)	37.26 (3.41)	0.93	.339	.02
* Integrate emotion to facilitate thought*	34.86 (4.98)	38.96 (4.42)	**8.53**	**.006**	**.17**
* Use of emotions*	33.55 (5.42)	36.04 (3.96)	3.14	.084	.07
* Regulation of emotions*	32.86 (5.74)	37.83 (4.89)	**9.78**	**.003**	**.19**
IRI					
* Perspective taking*	18.59 (4.27)	20.13 (4.16)	1.50	.227	.03
* Fantasy*	18.14 (4.50)	18.35 (5.91)	0.02	.893	.00
* Empathy concern*	18.59 (4.08)	19.35 (4.27)	0.37	.547	.01
* Personal distress*	13.82 (4.16)	7.96 (4.28)	**21.68**	**<.001**	**.34**
BDHI	45.32 (9.33)	41.61 (8.60)	1.93	.172	.04
BIS	67.77 (9.24)	58.96 (8.93)	**10.60**	**.002**	**.20**

mean (SD); bold: statistically significant.

RMET, Reading the Mind in the Eyes Test; EQ, Emotional Quotient; IRI, Interpersonal Reactivity Index; BDHI, Buss-Durkee Hostility Index; BIS, Barratt Impulsiveness Scale-11-Revised.

The MANOVA results indicated significant group differences in alexithymic male and non-alexithymic male groups (Wilks’ lambda = 0.35, *F*_(12,32)_ = 4.85, *P* <.001, *η^2^_p_* = .65). Alexithymic male subjects showed poorer performance than male controls in the RMET (*F*_(1,43)_ = 4.55, *P* = .04, *η^2^_p_* = .10). Regarding self-questionnaires, there was a significant decrease in EQ perception and expression of emotion (*F*_(1,43)_ = 18.55, *P* <.001, *η^2^_p_* = .30), EQ integration of emotion to facilitate thought (*F*_(1,43)_ = 8.53, *P* = .01 *η^2^_p_* = .17), and EQ regulation of emotions (*F*_(1,43)_ = 9.78, *P* = .00, *η^2^_p_* = .19) for highly alexithymic males. They also reported both higher IRI personal distress (*F*_(1,43)_ = 21.68, *P* <.001, *η^2^_p_* = .34) and impulsivity than male controls (BIS score, *F*_(1,43)_ = 10.60, *P* = .00, *η^2^_p_* = .20).

#### Alexithymic Female Group vs. Non-Alexithymic Female Group

To obtain a statistical distribution similar to the male group within the female group, we contrasted the behavioral and clinical measures of only those participants who scored in the highest 25^th^ percentile (≥ 58 total TAS-20K score, *n* = 35) versus the lowest 25^th^ percentile (≤ 42 total TAS-20K score, *n* = 29) on the TAS-20K within the female group by conducting a MANOVA (dependent variables: RMET, EQ, IRI, BDHI, and BIS scores) (**Table 7**).

**Table 7:** Comparison of measurements in alexithymic and non-alexithymic female participants.

	Alexithymic(*n* = 29)	Non-alexithymic(*n* = 35)	*F*	*P*	*η_p_^2^*
*[Wilk’s lambda = 0.46, **F_(12,51)_ = 4.90, P <.001**, η^2^_p_ = .54]*					
RMET	26.07 (2.81)	26.51 (2.87)	0.39	.536	.01
EQ					
* Perception and expression of emotion*	34.03 (5.24)	39.86 (3.35)	**28.96**	**<.001**	**.32**
* Empathy*	37.31 (5.34)	38.57 (5.94)	0.78	.380	.01
* Integrate emotion to facilitate thought*	33.86 (5.43)	37.09 (4.42)	**6.87**	**.011**	**.10**
* Use of emotions*	33.83 (4.12)	37.11 (3.90)	**10.71**	**.002**	**.15**
* Regulation of emotions*	29.93 (4.96)	33.63 (4.96)	**8.81**	**.004**	**.12**
IRI					
* Perspective taking*	17.52 (3.62)	18.94 (5.09)	1.60	.210	.03
* Fantasy*	16.62 (5.72)	18.86 (5.74)	2.42	.125	.04
* Empathy concern*	17.72 (3.35)	18.63 (5.44)	0.61	.438	.01
* Personal distress*	18.24 (4.15)	12.60 (5.11)	**22.85**	**<.001**	**.27**
BDHI	46.31 (11.36)	46.11 (10.94)	0.00	.944	.00
BIS	67.59 (11.89)	61.86 (10.17)	**4.32**	**.042**	**.07**

mean (SD); bold: statistically significant.

RMET, Reading the Mind in the Eyes Test; EQ, Emotional Quotient; IRI, Interpersonal Reactivity Index; BDHI, Buss-Durkee Hostility Index; BIS, Barratt Impulsiveness Scale-11-Revised.

There were significant group differences in alexithymic and non-alexithymic females according to the MANOVA test (Wilks’ lambda = 0.46, *F*_(12,51)_ = 4.90, *P* <.001, *η^2^_p_* = .54). We found that, within females, highly alexithymic subjects reported attenuated emotional intelligence (EQ perception and expression of emotion: *F*_(1,62)_ = 28.96, *P* <.001, *η^2^_p_* = .32; EQ integration of emotion to facilitate thought: *F*_(1,62)_ = 6.87, *P* = .01, *η^2^_p_* = .10; EQ use of emotions: *F*_(1,62)_ = 10.71, *P* = .00, *η^2^_p_* = .15; and EQ regulation of emotions: *F*_(1,62)_ = 8.81, *P* = .00, *η^2^_p_* = .12). In contrast, scores on the IRI personal distress (*F*_(1,62)_ = 22.85, *P* <.001, *η^2^_p_* = .27) and BIS (*F*_(1,62)_ = 4.32, *P* = .04, *η^2^_p_* = .07) were increased in the alexithymic group compared with the female control group. Intriguingly, there was no evidence of poor performance on the RMET in alexithymic female subjects (*F*_(1,62)_ = 0.39, *P* = .54, *η^2^_p_* = .01).

## Discussion

In the past decade, alexithymia, a multifaceted construct, has been an attractive topic in clinical research (68). However, previous findings were not valid enough to ensure a reliable exploration of the adverse effects of alexithymia because of methodological issues and sample heterogeneity (16, 69, 70). In the present work, we recruited participants from the general population to perform representative alexithymia assessments and the RMET to measure emotion recognition ability and then examined not only the clinical and behavioral features observed in alexithymia but also the gender-specific alexithymic phenotypes.

First, we found that self-reported difficulties in emotion recognition, integration, and regulation were salient for alexithymic participants compared to non-alexithymic controls, regardless of gender variance. Intriguingly, there were no significant reductions in most empathy measures for alexithymia. Rather, IRI personal distress scores measuring the level of emotional distress or reactivity in stressful situations were significantly increased in the alexithymia groups compared to the control groups.

We also completed multivariate analyses to identify the discriminative gender-specific features of alexithymia. As a result, symptom severity of alexithymia was shown to be greater in females than in males, which is consistent with previous findings using general populations (e.g., university students) (6, 42, 43). Additionally, even though the highly alexithymic females reported themselves as having difficulties in emotion processing, the RMET performance was preserved according to the within-gender analyses. The male participants with high alexithymia scores, who self-reported functional impairments in most areas of emotional intelligence except emotion utilization, revealed increased impulsivity as well as poor RMET performance. All findings were replicated *via* nonparametric statistical analyses (Kruskal-Wallis *H* test), and the following subsections provide more detailed descriptions.

### Emotion Recognition (TAS-Difficulty Identifying Feeling, EQ-Emotion Perception and Expression, RMET)

Compared to the participants with little alexithymia, highly alexithymic individuals revealed increased subjective difficulties in emotion processing in accordance with previous research (48, 69). However, the behavioral experiment using the RMET showed different patterns according to gender. Alexithymic female subjects were found to maintain the ability of emotion recognition, while compared to non-alexithymic males, alexithymic males exhibited poor performance on the RMET compared to non-alexithymic males. Given previous reports on the preserved ability to decode facial expression in alexithymia (22, 23) as well as the female superiority over males on the RMET (71, 72), this gender difference seems to be a reasonable finding.

In particular, the finding of intact RMET performance in females is consistent with the results of a recent alexithymia study showing a normal performance of judgment for facial emotion blends (23). However, even with intact performance on average, emotional stimuli seemed to be challenging for alexithymic individuals; the alexithymic participants had a reduced viewing preference for facial eye regions. Looking at the eyes of the blended faces even increased performance errors. According to the authors, their findings reflected intolerance of intense emotion or attempts to avoid emotional stimuli (22) in alexithymic individuals.

The fragility of RMET performance related to alexithymia in males is revealed in the additional correlation analyses. In the case of male subjects, consistent with previous studies (11, 73, 74), alexithymia measured by TAS-20K appeared to be negatively correlated with an impaired ability to decode emotional experience as assessed by the RMET (TAS-20K total score *r* = −.25, *P* <.05; TAS-20K DIF factor *r* = −.29, *P* <.001; see **Table 4**). These correlations were not observed in the female subjects (**Table 5**). There has been a report that individuals with the most pronounced subjective difficulties in identifying feelings are more likely to have psychiatric symptoms (75). In addition to subjective discomfort, declined social cognition may pose further distress to social relationships among alexithymic individuals.

### Emotion Regulation (EQ-Emotion Regulation, BDHI, and BIS)

One factor that makes the daily functioning of individuals with high alexithymia scores even more difficult is their inefficient manner of regulating negative emotion (76). Generally, subjective difficulties in emotion regulation are related to undifferentiated and unidentifiable negative moods (77). Along these lines, prior research has suggested possible links among alexithymia, emotion dysregulation, and impulsivity (32).

In our study, impulsivity was found to be prominent in highly alexithymic individuals. In this study, subjective difficulties in toning down emotional arousal were also markedly reported in the current alexithymic participants. The larger effect size in the male group (*η_p_^2^* = .20) than in the female group (*η_p_^2^* = .07) seems to reflect gender-related emotion processing. For instance, it has been suggested that female genders are more likely to ruminate over a variety of issues. In contrast, males may take action explicitly concerning these issues (78). In particular, alexithymic males who show unstable ability to regulate their emotions may easily engage in impulsive aggression (79) as a way to inappropriately vent their emotions (80). Of note, the impulsivity of male subjects was found to be closely related to high scores on the TAS-20K EOT factor (*r* = .49, *P* <.001; see **Table 4**), which has a negative correlation with EQ regulation of emotions (*r* = −.38, *P* <.001). The above results were not prominent in the female subjects. This result is in line with the findings of a previous study carried out by Lander, Lutz-Zois, Rye, and Goodnight (81). They showed that psychopathy was correlated with the TAS-20K EOT factor, representing a cognitive tendency to focus on one’s external rather than internal world. Indeed, an EOT style is related to social detachment and impaired cognitive processing (82). Thus, further research regarding the finely dimensioned profiling of alexithymia was necessary to uncover the underlying mechanisms responsible for alexithymia-related outcomes.

However, unexpectedly, there was no significant group difference in hostility, even though there was a positive relationship between BDHI and TAS-20K scores in male subjects. This seems to be because the scale used in this study reflects only direct and active hostility (59). Given that previous studies have reported a link between hostility and alexithymia (28), further study needs to identify the dynamics of alexithymia using a tool that can measure passive aggression, which involves the use of implicitly hostile words or behaviors as well as active forms.

### Empathy (EQ Empathy, IRI Subscales)

Although many empirical findings have attempted to define a link between alexithymia and an empathy deficit (73, 80), the evidence for this association is quite obscure (11).

In the current general population, alexithymic male and female subjects appeared to show no deficits in most empathy measures, except for the personal distress scale of the IRI, relative to their respective gender control subjects. Rather, the scores on the personal distress scale were increased in both gender groups with highly alexithymic features. Correlation analyses also supported the following relationship between alexithymia and empathic over-arousal (83, 84): a higher tendency to suffer emotional distress in a stressful environment was associated with a higher level of alexithymia (males, *r* = .52, *P* <.001; females, *r* = .46, *P* <.001; see **Tables 4** and **5**).

Indeed, there have been reports on the strong evidence of coupling between alexithymia and greater emotion distress (85–87), which is correlated with high emotionality (56). Beadle and colleagues (85) noted that observing others’ sufferings may lead to high levels of negative arousal in those who try to control their emotions in the absence of proper emotion regulation ability. Thus, it has been suggested that alexithymic individuals should disguise their negative feelings in order to protect themselves from aversive and overwhelming emotional experiences (20, 21).

This finding reminds of the concept of “secondary” alexithymia depicted by McDougall (18) as a defense mechanism. Some authors were interested in exploring the existence of two types of alexithymia (88–90): type I, associated with little emotional arousal (affective alexithymia dimension, i.e., schizoid personality trait), and type II, in which emotion lability is present but disconnected from effective emotion regulation at the higher cognitive level (cognitive alexithymia dimension, i.e., borderline personality trait) (90, 91). According to this perspective, the observed features of alexithymia in our general population may be classified as type II, cognitive alexithymia. Indeed, since the TAS-20, which is used in this study, is currently regarded as reflecting the cognitive alexithymia dimension (90), this pattern of results seems plausible. Further empirical investigations adopting various screening tools are needed to illuminate the dominant type in the general population.

### Limitations

Even though the current samples seem appropriate to reveal the characteristics of alexithymia [mean TAS-20K score of 59.30 ± 5.82 regarding the observed level of alexithymia in previous studies (e.g., mean TAS-20 score of 54.10 ± 1.49 by Berenson et al. (92) and 58.60 ± 12.84 by Pluta et al. (93))], this study has several drawbacks. First, the participants were relatively young. A larger sample that includes older adults should be considered for further research. Future studies may also benefit from increasing the clinical measures considering depression and anxiety, which are common in the general population, to provide abundant clues to the phenotypes of alexithymia. Additionally, because this study had a cross-sectional design, causality could not be established. Longitudinal studies with larger samples will be helpful for clarifying the associations among alexithymia, empathic distress, and externalizations. Lastly, as the results of the present analyses do not allow us to propose the underlying mechanisms of alexithymia-related features; further research is thus needed to construct an experimental design regarding latent profile analysis, thought suppression and defense mechanisms (94, 95).

## Conclusions

The definition of alexithymia, which originally meant a deficiency in the ability to identify one’s own emotional state, was gradually extended to a subclinical state with a weakened ability to perceive others’ emotions or experience empathy. The purpose of this study was to examine the dominant features of alexithymia, focusing on emotion processing, empathic ability, hostility, impulsivity, and gender. In conclusion, alexithymic individuals demonstrated subjective difficulties in both emotion recognition and emotion regulation. We also found that alexithymia-related emotional frustration and personal distress may result in dysfunctional externalized expression such as impulsivity against unidentifiable and unpleasant emotion experiences. However, highly alexithymic females appear to underestimate their own ability to perceive the emotional status of others relative to their actual performance on an emotion recognition task. An interesting finding was that alexithymic individuals in this study revealed preserved empathy skills and even exhibited an increased proneness to experience empathic over-arousal with others’ suffering. Although empathy generally enhances our social connectivity, it is also true that a higher level of empathic distress has detrimental effects on efficient emotion processing for those who lack the ability to manage their own highly aversive feelings in response to others’ suffering. As a whole, these results suggest that those who are highly susceptible to negative emotional stimuli may avoid engaging with emotional experiences that they find disturbing and, especially in females, may underestimate their ability for emotion processing. Further research efforts should focus on the development of new nosological strategies to elucidate the underlying mechanism of alexithymia regarding actual emotion recognition ability, empathic distress, as well as gender factors.

## Data Availability Statement

The original contributions presented in the study are included in the article/supplementary material; further inquiries can be directed to the corresponding author.

## Ethics Statement

The studies involving human participants were reviewed and approved by Chung-Ang University Ethics Committee. The patients/participants provided their written informed consent to participate in this study.

## Author Contributions

GN: conceptualization, formal analysis, methodology, visualization, writing—original draft, writing—review and editing. J-WH: conceptualization, formal analysis, methodology, visualization, writing—original draft, writing—review and editing, funding acquisition, project administration. HL: data curation, investigation. J-HL: resources, supervision.

## Funding

This research was supported by the Brain Research Program of the National Research Foundation (NRF) funded by the Korean government (MSIT) (NRF-2017M3C7A1048040).

## Conflict of Interest

The authors declare that the research was conducted in the absence of any commercial or financial relationships that could be construed as a potential conflict of interest.
